# High frequency of benzimidazole resistance alleles in trichostrongyloids from Austrian sheep flocks in an alpine transhumance management system

**DOI:** 10.1186/s12917-020-02353-z

**Published:** 2020-05-11

**Authors:** Barbara Hinney, Julia Schoiswohl, Lynsey Melville, Vahel J. Ameen, Walpurga Wille-Piazzai, Karl Bauer, Anja Joachim, Jürgen Krücken, Philip J. Skuce, Reinhild Krametter-Frötscher

**Affiliations:** 1 Department of Pathobiology, Institute of Parasitology, Vetmeduni Vienna, Veterinärplatz 1, 1210 Vienna, Austria; 2 Department for Farm Animals and Veterinary Public Health, University Clinic for Ruminants, Vetmeduni Vienna, Veterinärplatz 1, 1210 Vienna, Austria; 3 Moredun Research Institute, Pentlands Science Parks, Bush Loan, Penicuik, Edinburgh, EH26 OPZ UK; 4 Institute of Parasitology and Tropical Veterinary Medicine, Freie Universität Berlin, Berlin, Germany; 5 College of Veterinary Medicine, University of Duhok, Duhok, Kurdistan Region Iraq; 6 Animal Health Service Styria, Friedrichgasse 11, 8010 Graz, Austria

**Keywords:** Nematode, Anthelmintic resistance, Single nucleotide polymorphism, Trichostrongyloids, pyrosequencing

## Abstract

**Background:**

Infections of small ruminants with trichostrongyloid nematodes often result in reduced productivity and may be detrimental to the host. Anthelmintic resistance (AR) against most anthelmintic drug classes is now widespread amongst the trichostrongyloids. Baseline establishment, followed by regular monitoring of the level of AR, is necessary for farmers and veterinarians to make informed decisions about parasite management. The detection of single nucleotide polymorphisms (SNPs) is a sensitive method to detect AR against benzimidazoles (BZs), one of the most widely used anthelmintic classes. Alpine transhumance constitutes a special type of pasturing of sheep from many different farms, the aim of this study was to investigate the prevalence of benzimidazole resistance alleles in this particular management system.

**Results:**

Sixteen sheep flocks in Styria and Salzburg in Austria were examined by pyrosequencing for SNPs at codons 167, 198 and 200 of the isotype-1 β-tubulin gene. The frequency of the resistance-associated exchange F200Y was 87–100% for *H. contortus,* 77–100% for *T. colubriformis* and <  5–66% for *T. circumcincta*. Additionally, the F167Y polymorphism was detected in *T. colubriformis* from two farms at a frequency of 19 and 23% respectively.

**Conclusions:**

The high resistance allele frequency in *H. contortus* and *T. colubriformis* in the examined sheep population urgently calls for the development of new treatment strategies to sustainably control trichostrongyloid infections for this kind of pasturing, since the frequent mixing of flocks during the alpine summer grazing must be considered an important risk factor for the spread of resistant nematodes to a large number of farms.

## Background

Transhumance, the seasonal movement of livestock between mountain and lowland pastures, is a common form of pasturing in alpine areas. There are only few studies investigating the influence of this form of pasturing on infections with gastrointestinal nematodes (GIN) in sheep and on the development of anthelmintic resistance (AR), and GIN-related problems are barely characterized in this management system. Nevertheless, there are indications that common characteristics of mountain farming might promote the development of AR [1]. As GIN infections of small ruminants significantly affect animal health and thus farm profitability [2–5], widespread AR could seriously impair the current form of alpine sheep farming. Trichostrongyloids are the most prevalent nematodes of sheep, with *Trichostrongylus colubriformis, Haemonchus contortus* and *Teladorsagia circumcincta* amongst the most important. Infection with these parasites can result in reduced productivity of animals through poor weight gain, weight loss, reduced wool and milk production as well as weakness or ill-thrift [6–8]. Infection may also result in sudden death, especially when *H. contortus* is involved, as this haematophagous species is particularly pathogenic [9, 10].

Recommendations for best practice control of GIN underwent significant changes during the last decades worldwide. Since the introduction of broad-spectrum anthelmintics with good tolerability, control measures relied heavily on frequent application of chemical anthelmintics to the whole flock [11]. This has resulted in the selection for AR in nematodes against the main groups of anthelmintics, the benzimidazoles (BZs), the imidazothiazoles/tetrahydropyrimidines and the macrocylic lactones (MLs) and across multiple GIN species [12–14]. Currently, AR against BZs is the most common form, but the prevalence of multi-drug resistance is increasing [15–17]. New anthelmintics such as derquantel and monepantel have been developed, however, resistance against monepantel has already been described only 2 years after its introduction [18]. Consequently, the rapid development and spread of AR makes modern parasite management increasingly challenging [4, 19, 20].

There is consensus that constant monitoring of the occurence of AR is required to maintain anthelmintic efficacy, as the early detection of AR is necessary for timely adjustment of parasite management measures, either in terms of treatment timing, product choice or both [8, 21].

For the detection of AR, the faecal egg count reduction test (FECRT) is the most commonly used method [22]. This in vivo test has the advantage of being applicable to all anthelmintic drug classes. However, it is labour intensive and only detects resistance if the level of genetically resistant individuals in the population is relatively high, above 25% [23]. In addition, the egg hatch test (EHT) or the larval development test are in vitro tests which have been standardised for several anthelmintics, but are also labour-intensive, and the EHT also does not detect low resistance levels [23–25].

By contrast, molecular tests have very high sensitivity and specificity for the early detection of non-synonymous single nucleotide polymorphisms (SNPs), which are useful genetic markers for resistance [24, 25]. Knowledge of the role of these SNPs and other genetic markers is only sufficiently characterized for BZs, so BZs are currently the only drug class where a SNP analysis can indicate a resistant phenotype. In BZ-susceptible nematodes, BZs bind specifically and with high affinity to the nematode isotype-1 β-tubulin and prevent polymerisation of tubulin dimers into microtubules. Impaired microtubule function results in a reduced glucose uptake and protein secretion [26, 27]. In resistant nematodes, mutations in the isotype 1 β-tubulin gene cause structural changes in the β-tubulin through amino acid changes that prevent BZs from binding [28]. These structural changes are associated with certain SNPs [29]. A SNP at codon 200 of the isotype 1 beta-tubulin gene which substitutes tyrosine (Tyr) for phenylalanine (Phe) (TTC > TAC) has been demonstrated in resistant isolates of *H. contortus, T. colubriformis* and *T. circumcincta* in sheep and is the most common SNP associated with BZ resistance [30, 31]. In addition, SNPs at two codons 167 and 198 (F167Y: TTC- > TAC; E198A: GAG- > GCG; E198L: GAG- > TTG) have also been correlated with BZ resistance but occurred in lower frequency [32–34]. Although not all cases of resistance can be explained by the presence of these SNPs, their frequency in a population is able to explain the vast majority of reported cases of BZ resistance. There is a high correlation between the percentage of resistance alleles and a reduced FECR or increased EC_50_ in the EHT. However, screening for these SNPs is currently considered to be the most sensitive, robust and effective approach to screen field samples for the presence of BZ resistance [25, 35–37]. Different molecular assays for the detection of SNPs have been developed. Pyrosequencing is amongst the most commonly used [24, 38]. For Austria, only very limited information is currently available on the status of AR in sheep nematodes [39]. Since knowledge about the efficacy of anthelmintic drugs is essential for the surveillance of AR development, we aimed to obtain data on BZ resistance in alpine regions of Austria, where grazing livestock, including sheep, is an integral part of agriculture.

The study presented here is part of a project on the surveillance of animal health in transhumance of sheep [40, 41]. As multi-drug AR is increasingly reported worldwide, it was one aim of the project to evaluate the efficacy for the most common anthelmintic drug classes. Due to the fact that no molecular markers for ML resistance are available, efficacy of moxidectin was evaluated by a FECRT and was shown to be reduced in vivo with one out of 16 farms showing resistance (egg count reduction of 93%) and two farms with suspected resistance (LCL below 90%) [40], stressing the importance of frequent AR monitoring. Using the material collected during the FECRT before treatment, the distribution and prevalence of BZ resistant genotypes in sheep from alpine transhumance flocks was assessed in the present study by pyrosequencing to provide additional information on the drug sensitivity of the nematodes infecting the examined sheep population.

## Results

Larval pools from the different farms contained 500 to 61,600 larvae (Table 1). Percentages of *H. contortus*, *T. circumcincta* and *T. colubriformis* in pooled larval samples were between 0 and 48%, between 2 and 22% and between14 and 67%, respectively (Table 1). *Bunostomum, Cooperia, Chabertia and Oesophagostomum* were also identified in pooled samples, at low prevalence. Detailed information about morphological differentiation of larvae is given in Schoiswohl et al. [41].

**Table 1:** Frequencies of alleles containing the F167Y, E198A and F200Y exchanges correlating with benzimidazole resistance for *Haemonchus contortus*, *Teladorsagia circumcincta* and *Trichostrongylus colubriformis.* (< 5% = below background threshold)

Farm	N larvae in pooled samples	% of trichostrongyloid species in pooled third stage larval samples used for pyrosequencing			***H. contortus*** % F200Y	***T. circumcincta*** % F200Y	***T. circumcincta*** % F167Y	***T. colubriformis*** % F200Y	***T. colubriformis*** % F167Y
		*Haemonchus*	*Teladorsagia*	*Trichostrongylus*					
1 (FT)^*^	1566	4	7	32	49; 71^a^	n.a.	< 5	100	< 5
2 (FT)^**^	17,600	48	2	36	96	66	< 5	99	< 5
3 (FT)^**^	**14,400**	**42**	**12**	**36**	**93**	**31**	**< 5**	**n.d.**	**n.d.**
4 (FT)^**^	**4666**	**26**	**17**	**40**	**95**	**n.a.**	**< 5**	**77**	**23**
5^*^	1600	36	5	39	87	18	n.a.	n.d.	n.d.
6^**^	500	0	8	66	n.a.	< 5	< 5	n.d.	n.d.
7^**^	8500	30	12	39	98	n.a.	n.a.	n.d.	n.d.
8^*^	61,666	21	2	42	n.a.	48	< 5	n.d.	n.d.
9^*^	**1666**	**1**	**6**	**67**	**99**	**n.a.**	**n.a.**	**97**	**< 5**
10 (FT)^**^	2000	39	8	50	100	11	n.a.	100	< 5
11^*^	2666	14	2	14	94	40	< 5	n.d.	n.d.
12 (FT)	42,666	16	8	53	n.a.	47	< 5	98	7
13 (FT)^**^	2666	2	22	58	100; 94^a^	31	< 5	95	9
14^*^	n.d.	11	9	36	n.a.	n.a.	n.a.	82	19
15 (FT)^*^	n.d.	3	17	61	n.a.	n.a.	< 5	100	6

*n.d.* No data (no DNA left for analysis); *n.a.* No amplicon generated; *FT* Full-time farms, all other farms were sideline enterprises (small-holder farms); ^*^: indicates farms that used dewormers once a year; ^**^: indicates farms that used dewormers two times a year; **bold letters**: farms where a reduced efficacy of moxidectin had been observed; ^a^Two samples were examined

Codon 167 was successfully analysed in *T. colubriformis* and *T. circumcincta* in nine and ten farm populations respectively. Codons 198 and 200 were analysed in *H. contortus*, *T. colubriformis* and *T. circumcincta* from ten, nine and nine of the 16 farm populations, respectively. Data for all five SNPs was obtained from two farms. Missing data was due to failure to produce PCR amplicon for pyrosequencing analysis (detailed in Table 1 as ‘n.a.’) or where insufficient DNA was available to run pyrosequencing assays for all SNPs of interest (detailed in Table 1 as ‘n.d.’).

The F200Y exchange was identified in all ten of the *H. contortus* populations tested; mean frequency of resistance alleles 91.9 ± 3.7% (mean ± SEM), range 49–100%. In *T. circumcincta*, F200Y was identified in eight of nine populations analysed at a mean resistance allele frequency of 32.4 ± 6.8%, range 0–66%. No resistance alleles were observed at codon 167 of *T. circumcincta* in any of the populations analysed. The F200Y exchange was identified in all nine *T. colubriformis* populations tested; mean resistance allele frequency 94.2 ± 2.9% (range 77–100%), F167Y was identified in five of the nine populations tested; mean resistance allele frequency 7.1 ± 2.9% (range 0–23%) (Table 1). Results for codon 198 for all three species revealed no BZ resistance-associated alleles (frequencies below the technical background threshold of 5%).

### Farm structure and deworming practices

Eight farms were full-time farms and seven were sideline enterprises (small-holder farms) (Table 1).

For 14/16 farms, information on deworming frequencies was available (Table 1). Seven farms used dewormers two times a year (in spring and autumn); the other seven farms used dewormers only once a year in spring.

For three farms, a reduced efficacy of moxidectin had already been observed [40]. No visible or significant difference on the percentage of resistance alleles was observed between farms for any of these variables.

## Discussion

This is the first study evaluating for the presence of resistance alleles in ovine trichostrongyloids in Austria, focusing on sheep flocks within a transhumance management system. No FECRT could be performed because after several reports of treatment failure with BZs from attending veterinarians, farmers were not willing to use BZs to deworm their sheep. Consequently, all study animals were treated with moxidectin. The combination of FECRT for ML and molecular tests for BZs allowed estimation of resistance status regarding both drug classes in parallel. While it may be argued that the SNP-analysis is only an indirect diagnosis of resistance, there is convincing evidence available that the percentage of resistance alleles containing the exchanges F167Y, E198A or F200Y strongly correlates with the results of the FECRT and/or the EHT [24, 35, 37, 42, 43] while susceptible populations have very low levels of these resistance alleles. We also want to highlight that FECRT and EHT can even lead to inconclusive results in field populations with mixed infections [44]. Thus, by analysing specifically a single species, molecular analyses can overcome the limitations of in vivo and in vitro tests with mixed field samples.

Although all three SNPs have been reported to be associated with AR to the benzimidazoles, codon 200 remains the most prevalent and arguably the most important [25].

The F200Y exchange was found to be highly prevalent in *H. contortus* and *T. colubriformis* on the tested farms. *T. circumcincta* populations generally had lower F200Y allele frequencies, greater than 40% in five of the nine populations tested. A recent study identified the F200Y exchange at frequencies up to 13% in benzimidazole-susceptible strains of *T. circumcincta*, while resistant strains had allele frequencies above 60% [43]. This suggests that phenotypic benzimidazole resistance could be possible in some of the *T. circumcincta* populations tested within this study. The interaction between the F200Y and F167Y polymorphisms is not fully understood however, it has been suggested for *T. circumcincta* that F200Y mutation may predominate initially with the frequency of F167Y increasing with phenotypic resistance [45]. From the results presented, it could be hypothesised that BZ-resistance may be at an advanced stage in *T. colubriformis* in the region tested. Unfortunately, due to limited sample material, we were not able to perfom the SNP analysis on codon 167 (F167Y) of *Haemonchus contortus* although this SNP remains an important polymorphism in other regions of the world [24, 25]. However, the nearly complete manifestation of F200Y in the *Haemonchus-*samples suggests a high prevalence of clinically relevant BZ resistance.

Thus, it can be concluded that a high percentage of BZ resistant *H. contortus* and *T. colubriformis* are present in sheep in this region of Austria, while the frequency of resistant *T. circumcincta* remains moderate. This also supports anecdotal reports of poor efficacy of BZs in sheep in the investigated area. When compared to recent data [25], the frequency of resistance alleles of *H. contortus* in Austria is amongst the highest in Europe. This may be due to a long-standing “dose and move” practice on many Austrian farms practicing transhumance. Mutations leading to AR can arise due to the very large worm population sizes and be rapidly enriched due to strong selection by frequent strategic treatments in local nematode populations [34].

Similar high levels of BZ resistance were observed in goats in the alpine region of Italy [1, 46] supporting the assumption that resistance alleles associated with AR were spread through the introduction of resistant strains via animal movement and communal high altitude grazing. Dose and move strategies and animal movement are also common in Styria and Salzburg [41] and might be an important driving factor for the spread of AR on all farms investigated. In addition, strategies to maintain a refugium of anthelmintic-susceptible parasites, known to reduce the rate of development of anthelmintic resistance in GIN [11], may be especially difficult to implement when animals from different farms and management strategies are herded together on communal pastures. The deworming frequency in the study populations was low (1–2 treatments per year), but the deworming of all animals in spring before they are turned out to the mountain pastures clearly reduces the refugium for susceptible worms.

*Haemonchus* sp. are sensitive to cold weather [47], and few *Haemonchus* larvae will survive the harsh conditions of the alpine winter. Consequently, *Haemonchus* overwinters largely in hypobiosis within the host, which accounts for the high prevalence observed in some regions with northern boreal/continental climate [25, 35, 47, 48]. When all animals are treated before winter turn-in, *Haemonchus* encounters a significant bottleneck for survival [49], which increases the selective advantage for resistant worms that overwinter in their host and survive spring treatment, quickly increasing the proportion of resistant individuals on summer pastures.

Considering the present results in combination with the high fecundity of *Haemonchus* [47], there is considerable concern that the population of BZ resistant *Haemonchus* in Austria may expand further in size and geographic range e.g. through treatment errors, animal movement and/or the spread by wildlife hosts [50]. Indeed, *Haemonchus* increased in relation to other trichostrongyloids in faecal cultures examined during the course of the grazing season [41]. This trend was independent of BZ treatment as the sheep involved in the study were only treated with moxidectin during the investigated time period. It should, however, be mentioned that also macrocyclic lactone treatment might select for the variants F200Y and F167Y of *Haemonchus* [51].

One of the most popular strategies to slow the rate of development of AR is targeted selective treatment [20]. This leaves selected animals untreated. Untreated animals will then shed eggs of worms that were not exposed to anthelmintics, so presumably susceptible to the AH. These eggs of untreated animals will dilute eggs produced by any resistant worms which survived treatment within treated animals, and thus ameliorate the strong effects of drug selection by maintaining susceptible parasites in refugia [11, 20, 47]. However, this strategy requires that a sufficient proportion of alleles associated with a susceptible phenotype are present within the population. The results of the present study suggest a lack of susceptibility-associated alleles at codon 200 in the *H. contortus* populations. Therefore, leaving some sheep untreated would not be effective in reducing resistance allele frequencies and thus BZ resistance, unless a susceptible population of *H. contortus* was introduced [52]. Ideally, susceptibility of this introduced worm population should be tested at the genetic level. It has also to be considered that adding only *H. contortus* larvae into the population would increase the relative prevalence of this highly pathogenic species in the overall trichostrongyloid population potentially resulting in greater production losses, so until more evidence of this strategy is provided we would not recommend this approach. Nevertheless, selective treatment is still considered to be an appropriate strategy for the region under investigation in this study, especially for slowing down selection of AR against other anthelmintic classes.

The fact that the prevalence of BZ resistance genotypes was very high in three flocks where ML resistance was also observed shows that multi-drug-resistant trichostrongyloid nematodes of sheep can also occur in extensive management systems such as transhumance in alpine Austria. If no alternative strategies are implemented in the near future, it must be expected that AR will be widespread and irreversible [53]. Information and instruction to farmers by veterinarians is strongly recommended, especially since a considerable part of the Austrian sheep population is kept by farmers with sideline enterprises or hobby farmers who may not have sufficent knowledge and experience with novel antiparasitic management.

In the longer term, economical and sustainable management of grazing livestock will likely depend on three main strategies: (1) rational use of licensed anthelmintic drugs to slow down the selection of resistant populations using approaches such as targeted selective treatment [20], (2) the development of new effective anthelmintic drug classes and (3) the improvement of strategies that do not depend on the administration of anthelmintics [54]. For the latter there are a number of approaches such as vaccination, breeding and grazing management. Vaccination against haemonchosis is a promising development for farms heavily afflicted by this worm [55–57]. The first commercial vaccine, Barbevax®, has been evaluated in several countries and is available in Australia since 2014 [58]. However, the high frequency of vaccination required in the first 2 years would be very difficult to implement under transhumance farming management. Animals kept in transhumance are not easely accessible and this will intensify the work load for farmers tremendously. Breeding sheep that are resilient or resistant to worm infections is another feasible strategy [59] and appropriate grazing management is still the cornerstone of non-chemical strategies [47, 60].

## Conclusion

In agreement with recent studies from many geographical regions, a high frequency of AR genotypes in nematodes from Austrian sheep kept in transhumance husbandary systems was detected. Management and climate factors in mountain regions might increase the rate of selection for resistant worms by reducing refugia on pasture. Furthermore, the joint grazing of animals from many different farms promotes gene flow and the development of AR. In order to preserve productivity of sheep farming in this area in future, there is an urgent need to implement alternative strategies in transhumance that do not rely so much on the use of anthelmintics as current management practices and that help to maintain refugia in alpine grazing systems.

## Methods

### Collection of field samples

The collection of field samples was published previously [40, 41]. Briefly, 243 adult animals (ewes) from 16 farms (6–31 animals/farm) in the federal states of Styria (*n* = 13) and Salzburg (*n* = 3), Austria, were examined. These sheep were stabled over winter and brought to pens near to the farms in spring before they were moved to alpine pastures together with sheep from other farms. Faecal samples used for this study were taken in March 2015, shortly before turnout to communal pastures. The farms that were included were attended by the the veterinary animal health service advising the best strategy for anthelmintic treatment. All animals were treated with moxidectin because it was considered to be the most effective anthelmintic drug. The history of anthelmintic drug application was documented in as much detail as possible, as described in [41].

### Larval cultures

For each farm, one pooled sample of third stage larvae was obtained by faecal culture and morphologically speciated as described previously [61]. Larvae where exsheathed in an aqueous solution containing 5% (v/v) sodium hypochlorite, washed three times in distilled water followed by centrifugation at 150 rpm for 10 min and maintained in 70% (v/v) ethanol at 4 °C until further processing.

### DNA extraction

Before extraction, samples were allowed to sit for 15 min at 35 °C to allow ethanol to evaporate. Then, DNA was extracted from whole pools of 500 to 61,600 larvae (Table 1) using the NucleoSpin® Soil Kit (Macherey-Nagel, Düren, Germany) following the manufacturer’s instructions, using buffer SL1 without enhancer. The DNA was eluted in 100 μl of the elusion buffer (SE) and the DNA samples were stored at − 20 °C until use.

### Determination of SNP frequencies

Pyrosequencing assays for the detection SNPs leading to the exchanges E198A and F200Y in both *H. contortus* and *T. circumcincta* and F167Y in *T. circumcincta* were carried out as previously described [24, 62]. Primer information is given in Table 2.

**Table 2:** Description of primers used

Targeted trichostrongyloid species; primer name	Sequence 5′ to 3′	Size [base pairs]	Tm [°C]	Reference
*Haemonchus contortus*				
HcPy2PCR For	GAC GCA TTC ACT TGG AGG AG	400	53	[24]
HcPy2PCR Rev	Biotin-CAT AGG TTG GAT TTG TGA GTT			
*Teladorsagia circumcincta*				
F200Y_FOR	Biotin- ACC TTA CAA TGC CAC TCT TTC TG	100	58	[62]
F200Y_REV	GCG GAA GCA GAT ATC GTA CAG			
F167Y_FOR	GCA TTC TTT GGG AGG AGG TA	200	58	
F167Y_REV	Biotin-TGC ACC TCG AGA ACC TGT ACA TA			
*Trichostrongylus colubriformis*				
FOR	TAC CCA GAT CGG ATT ATG TCT TC		58	[25]
REV	Biotin – GGC AAG TCG TGA CAC CAG ACA			
**Pyrosequencing primer**				
Hc200PySeq1 (*Haemonchus*, Codon 200)	TAG AGA ACA CCG ATG AAA	Not applicable		[24]
T. circ. F200Y_SEQ (*Teladorsagia*, Codon 200)	RGA GCY TCA TTA TCG ATR			[62]
T. circ. F167Y_SEQ (*Teladorsagia*, Codon 167)	CGG ATA GAA TCA TGG CT			[62]
*T. colubriformis* Codon 198_200	GCA GTA CTC GTG TC			[25]
*T. colubriformis* Codon 167	CGG ATA GAA TCA TGG CT			

Abbreviations

*AR* Anthelmintic resistance*BZ* Benzimidazole anthelmintics*GIN* Gastrointestinal nematodes*SNP* Single nucleotide polymorphism*ML* Macrocyclic lactones*FECRT* Faecal egg count reduction test*EHT* Egg hatch test

The PCR was conducted in 50 μl reaction volumes using the NovaTaq™ Hot Start PCR Master Mix Kit (Merck Millipore, Darmstadt, Germany). Each reaction contained 25 μl mastermix with a final MgCl_2_ concentration of 1.5 mM, 0.2 μM biotinylated primer, 0.4 μM of the non-biotinylated primer and 4 μl of template DNA. Using an Eppendorf MasterCycler Pro® (Eppendorf, Hamburg, Germany), reactions were incubated at 95 °C for 7 min followed by 45 cycles of 94 °C for 30 s, 58 °C (*T. circumcincta*) or 53 °C (*H. contortus*) for 30 s, 72 °C for 45 s and a final hold at 72 °C for 10 min. Five microliters of each PCR product were run on a 2% agarose gel, stained with Midori Green® DNA Stain (Nippon Genetics; Biozym, Oldendorf, Germany), subjected to vertical electrophoresis at a constant voltage of 120 V and scanned under UV light in a LumiBIS® (DNR Bio-Imaging System, Neve Yamin, Israel) to verify successful amplification. 45 μl of biotinylated PCR product were then used in each pyrosequencing assay.

Pyrosequencing reactions were performed targeting SNPs 167 and 198/200 in *T. circumcincta* and SNPs 198/200 in *H. contortus* using a PyroMark ID Pyrosequencer (Biotage, Uppsala, Sweden) following the manufacturer’s recommended protocol.

*T. colubriformis* assays were performed as described by Ramünke *et. al.* [25]. In brief, PCRs were conducted using Phusion® Hot-Start II High Fidelity DNA polymerase (Thermo Scientific, St. Leon-Rot, Germany). Reactions (50 μl) contained 200 mM dNTPs (Thermo Scientific), 250 nM of each primer, 1 U Phusion® polymerase and 4 μl genomic DNA. After an initial denaturation at 98 °C for 30 s, 40 cycles of denaturation at 98 °C for 10 s, annealing at a primer-pair specific temperature (Table 2) for 30 s and elongation at 72 °C for 30 s followed were performed before a final elongation at 72 °C for 10 min. PCRs were controlled by electrophoresis in 1.5% agarose gels stained with GR Green (Ozyme, Saint Cyr L’Ecole, France). Pyrosequencing assays were performed as previously described [63] using a PyroMark Q24 instrument and 22–45 μl of the PCR product.

For *T. circumcincta* and *H. contortus*, positive controls representing genomic DNA extracted from susceptible and resistant individual adults from each species were included in each assay. Assays for *T. colubriformis* were evaluated in separate runs using artificial mixtures of plasmid DNA containing the different SNPs as template. The frequencies of the resistance allele with values equal to or lower than 5% were considered as technical background and, therefore, not considered as resistant [37].

## Data Availability

The datasets generated and analysed during the current study are available from the corresponding author on reasonable request.

## Abbreviations

AR: Anthelmintic resistance
BZ: Benzimidazole anthelmintics
GIN: Gastrointestinal nematodes
SNP: Single nucleotide polymorphism
ML: Macrocyclic lactones
FECRT: Faecal egg count reduction test
EHT: Egg hatch test
